# Red Cell Phenotype Modifies the Association Between Free Triiodothyronine and Erythrocyte Count: A Cross-Sectional Study of 3673 Adults in a Hemoglobinopathy-Endemic Region of Saudi Arabia

**DOI:** 10.3390/diagnostics16213587

**Published:** 2026-10-30

**Authors:** Yahia A. Mjery, Saif Elden B. Abdalla, Tagwa Y. Elsayed, Edrous Alamer, Hazem Mathkour, Abdullah Farasani, Ali Baity, Fahd Alaajam, Faisal Madkhali, Nahla Kambal

**Affiliations:** 1 Department of Medical Laboratory Technology, College of Nursing and Health Sciences, Jazan University, Jazan 45142, Saudi Arabia; sebrahim@jazanu.edu.sa (S.E.B.A.); telsayed@jazanu.edu.sa (T.Y.E.); ealamer@jazanu.edu.sa (E.A.); hmathkour@jazanu.edu.sa (H.M.); aofarasani@jazanu.edu.sa (A.F.); abaity@jazanu.edu.sa (A.B.); falaajam@jazanu.edu.sa (F.A.); fmadkhali@jazanu.edu.sa (F.M.); 2 Clinical Nutrition Department, College of Nursing and Health Sciences, Jazan University, Jazan 45142, Saudi Arabia; nhmohammmed@jazanu.edu.sa

**Keywords:** triiodothyronine, erythropoiesis, erythrocyte indices, thalassemia, anemia, Saudi Arabia

## Abstract

**Background/Objectives**: Thyroid hormone regulates erythropoiesis, but most population studies have used thyrotropin (TSH) alone and have not examined whether the erythroid association differs by red cell phenotype. We examined this in a population where inherited microcytosis and iron deficiency are both common. **Methods**: We analyzed 3871 adults of both sexes with paired free triiodothyronine (FT3), free thyroxine, TSH, and complete blood count from three hospitals in Jazan, Saudi Arabia (3673 with FT3 within the reference interval formed the primary analysis). All three analytes were entered simultaneously in models adjusted for age and sex; mean corpuscular volume (MCV) and mean corpuscular hemoglobin (MCH) were pre-specified comparison (negative control) outcomes; red cell discriminant indices were used to assign a normocytic, acquired microcytic, or presumed inherited-trait pattern, which was compared against linked ferritin and transferrin saturation. **Results**: FT3 was associated with erythrocyte count (standardized β 0.097, 95% confidence interval (CI) 0.066–0.128, *p* < 0.001) and hemoglobin (+2.7 g/L per pmol/L); TSH was not (*p* = 0.547). The erythrocyte count association was present in normocytic (β 0.101) and acquired microcytic records (β 0.099) but absent in the presumed trait pattern (β −0.017; interaction *p* = 0.002) and remained absent under a stricter trait definition, whereas the hemoglobin association did not differ by phenotype (interaction *p* = 0.639). FT3 was unrelated to iron status, and the association survived adjustment for ferritin and restriction to same-episode iron measurements. In an exploratory comparison, both associations were strongest in iron-replete normocytic patients (hemoglobin +3.6 g/L per pmol/L, *p* < 0.001) and attenuated in measured iron deficiency. **Conclusions**: FT3, not TSH, was associated with erythrocyte count and hemoglobin. The count association was absent in the index-defined trait pattern, in which erythrocyte number is already high, and both associations were blunted where iron was limited. These observations are consistent with, but do not demonstrate, a limit on the marrow’s count response, since erythropoietic activity was not measured; they are hypothesis-generating and require replication with confirmed hemoglobin genotype, measured iron status, and reticulocyte or erythropoietin data.

## 1. Introduction

Thyroid hormone is a known regulator of erythropoiesis [1]. Triiodothyronine acts on erythroid progenitors through nuclear thyroid hormone receptors, accelerating their maturation and regulating the transcriptional program that governs hemoglobinization, including *GATA1*, *KLF1*, and the globin genes [2]. It is also required for terminal erythroid differentiation and enucleation, acting through thyroid hormone receptor beta together with the nuclear receptor coactivator NCOA4 [3]. An indirect route through erythropoietin has been proposed but is not well supported, since circulating erythropoietin appears largely independent of thyroid hormone concentrations [4]. Clinically, anemia frequently accompanies thyroid disease, and hemoglobin improves once thyroid dysfunction is corrected [5,6].

What remains unsettled is what happens across the physiological range, and in whom. Most clinical evidence compares overt thyroid disease against controls, a design that cannot separate a threshold effect of disease from a continuous relationship within the reference interval. Population studies have generally used thyrotropin (TSH) as the exposure, and the causal relationship between thyroid function and anemia has remained ambiguous [5]. Few have measured free triiodothyronine (FT3), free thyroxine (FT4), and TSH in the same individuals alongside a complete blood count, which is the minimum needed to ask which component of the axis carries an erythroid signal. One that did, the Busselton community cohort, found that FT3 and FT4 within the euthyroid range were positively related to hemoglobin and erythrocyte count, and that the FT3 relationship was curved rather than linear [7].

Genetic evidence has sharpened the question. A two-sample Mendelian randomization study in European populations found that genetically predicted FT4, regulated by variants in the deiodinase genes *DIO1* and *DIO2* [8], was causally associated with erythrocyte count, hemoglobin, mean corpuscular hemoglobin (MCH) and mean corpuscular volume (MCV), whereas euthyroid TSH was not [9]. If thyroid hormone influences erythropoiesis, the next question is whether the association is the same in everyone. One hypothesis is that it depends on what the marrow is able to change: where erythrocyte output is already high, or where hemoglobinization is limited by iron, the association may be weaker than where capacity is spare.

Answering that requires a population in which those constraints vary widely for reasons independent of thyroid status. The Jazan region of south-western Saudi Arabia provides such a setting. It is located in the Saudi belt of inherited hemoglobin disorders, where alpha thalassemia [10] and sickle cell trait [11] are common; both sickle cell disease and thalassemia are managed locally [12], and iron deficiency is also frequent. Microcytosis there arises from two mechanistically different causes, and erythrocyte count behaves oppositely in the two: thalassemia trait shows an increased erythrocyte count with small, poorly hemoglobinized cells, whereas iron deficiency reduces both. Saudi studies have reported a higher prevalence of anemia in thyroid dysfunction in the Asir region [13] and, in non-pregnant women in the Western province, correlations of erythrocyte indices with FT4 and of iron indices with TSH [14]; neither measured FT3 or considered red cell phenotype, and the relationship across the reference interval in a population with a high prevalence of inherited microcytosis has not been described.

We therefore examined whether FT3 is associated with erythrocyte count and hemoglobin independently of FT4 and TSH, whether that association is modified by red cell phenotype, and, using linked iron indices, whether it is modified by measured iron status. We hypothesized that any FT3 signal would be expressed through erythrocyte number where the marrow had capacity and would be weaker where it did not. The main finding was that FT3, but not TSH, was associated with erythrocyte count and hemoglobin and that the count association was absent in an index-defined, presumed inherited-trait pattern, and that both associations were blunted where hemoglobinization was iron-limited.

## 2. Materials and Methods

### 2.1. Study Design and Subjects

A cross-sectional study of routinely collected clinical laboratory data from three facilities in the Jazan region of Saudi Arabia: Baish General Hospital, King Fahad Hospital, and the Endocrinology and Diabetes Center. Data were extracted from the laboratory information system for the calendar year 2025. The export did not record the location from which each sample was collected. A separate analysis of thyrotropin in relation to the leukocyte differential, confined to Baish General Hospital records, is reported elsewhere; the present study asks a different question, the relation of free triiodothyronine to the erythron, for which concomitant free triiodothyronine and free thyroxine measurements were available only in the present cohort.

The study was conducted in accordance with the Declaration of Helsinki and approved by the Local Committee for Research Ethics, Jazan University (HAPO-10-Z-001; reference No. REC-46/09/1407, 4 March 2025) and the Jazan Health Cluster (Approval No. 2622). The requirement for individual informed consent was waived because the analysis used pre-existing, de-identified routine laboratory records.

Eligible records were those of patients with thyroid function testing and a complete blood count during the same laboratory episode, such that FT3, FT4, TSH, and a complete blood count were available. The export contained one record per patient identified by medical record number in the three facilities. We excluded records where sex was not recorded as male or female, patients less than 18 years of age, and records with an assay censoring flag on any thyroid analyte. The main analysis was restricted further to records where FT3 was within the assay reference interval, because of the functional form reasons given below.

### 2.2. Measurements and Variables

Complete blood counts were measured on Siemens hematology analyzers (Siemens Healthineers, Erlangen, Germany) and thyroid hormones by chemiluminescent immunoassay on Beckman Coulter Access analyzers (Beckman Coulter, Brea, CA, USA), both run under the manufacturers’ calibration and internal quality-control procedures. Records were categorized as euthyroid, subclinical or overt hypothyroid, or subclinical or overt hyperthyroid based on the reporting laboratory’s reference intervals (TSH 0.4–4.0 mIU/L, FT4 7.9–14.4 pmol/L, FT3 3.5–6.5 pmol/L); these intervals were consistent with the central 95% range observed in euthyroid participants in this cohort (FT4 6.8–14.1 pmol/L, FT3 3.8–6.6 pmol/L). The exposures were FT3, FT4, and TSH, the latter being natural-log transformed because of its strongly right-skewed distribution. All three were entered simultaneously into each model so that each coefficient represents that analyte’s independent contribution. The main endpoint was erythrocyte count.

MCV and MCH were pre-specified as negative control outcomes [15], that is, outcomes that share the confounding structure of the primary outcome but on which the exposure was not expected to act within the reference interval. The choice rested on two considerations. First, the confounders most likely to generate a spurious FT3–erythrocyte count association in routine data, namely chronic illness, undernutrition, hematinic deficiency, and thyroid hormone therapy, alter red cell size as well as number, so a confounded signal would be expected to appear on MCV and MCH as well as on count. Second, the primary hypothesis concerned erythrocyte number, and the size indices are measured on the same sample by the same analyzer, so they share its measurement and selection structure. We acknowledge that these are imperfect negative controls: Mendelian randomization has linked genetically predicted FT4 to MCH and MCV [9], and thyroid hormone contributes to hemoglobinization through thyroid hormone receptor beta and NCOA4 [3]. A null result on MCV and MCH therefore argues against gross confounding of the count association but does not exclude a direct effect of thyroid hormone on cell size, and we interpret these outcomes as a check on confounding rather than as proof of its absence.

Hemoglobin electrophoresis and iron studies were not part of the study protocol; iron indices were available only where they had been requested clinically (see below). Microcytosis was defined as MCV below 80 fL. Among microcytic records, a presumed inherited-trait pattern (hereafter “trait pattern”) was assigned when at least two of three red cell discriminant indices agreed: Mentzer index below 13, Green and King index below 65, and England and Fraser index below zero [16,17,18,19]. The remaining microcytic records were classified as an acquired pattern. Three indices were combined because each uses a different combination of MCV, erythrocyte count, hemoglobin, and red cell distribution width, and each has a different sensitivity and specificity for thalassemia trait against iron deficiency in meta-analysis [19]; a majority rule reduces dependence on any single cut-off. Records on which the indices disagreed (one of three positive) were assigned to the acquired pattern, which is the conservative direction for the primary hypothesis. A stricter definition requiring all three indices to agree was pre-specified as a sensitivity analysis. These indices are screening tools and do not identify a genotype, so the trait pattern is a laboratory phenotype that is enriched for, but not equivalent to, inherited hemoglobinopathy; it will include some iron-deficient records with a preserved erythrocyte count and will miss carriers with a normal MCV. The consequences of this misclassification for the stratified estimates are considered in the Discussion.

Iron status and other linked analytes. For validation of the red cell phenotype classification, serum iron, total iron-binding capacity, and ferritin results were linked by medical record number from the same laboratory information system for 2024 to 2025 (Baish General Hospital and the Endocrinology and Diabetes Center). Transferrin saturation was calculated as iron divided by total iron-binding capacity. Where several results existed, the median of the 2025 values was taken, or of the 2024 to 2025 values if none was available for 2025. The export records each patient’s order dates by calendar year but does not date individual results, so the interval between the index episode and the iron measurement could be fixed exactly only for patients with a single order date in 2025, for whom ferritin and the index tests were drawn in the same laboratory episode; for patients with several 2025 order dates, the interval could not exceed the span of those dates, and for patients whose only ferritin result was from 2024, the minimum interval was the time from their last 2024 order date to their first 2025 episode. We report the number of records in each category. Measured iron deficiency was defined as transferrin saturation below 16% or ferritin below 15 µg/L. Because iron studies are requested selectively, this linked subset is used only for validation and is not part of the primary analysis. As sensitivity analyses, the FT3 associations with erythrocyte count and hemoglobin were re-estimated in the same-episode subset and in the subset whose interval could not have exceeded 30 days, and, to address renal function and nutritional status, serum creatinine and vitamin B12 results from 2025 were linked in the same way (median of the year’s values) and entered as additional covariates; estimated glomerular filtration rate, folate, C-reactive protein, and reticulocyte count were recorded for too few patients to use. Hemoglobin electrophoresis was available for too few patients (*n* = 93) to validate the trait classification.

### 2.3. Statistical Analysis

Analyses were performed in Python 3.12 (Python Software Foundation, Wilmington, DE, USA) with pandas 3.0.2, NumPy 2.4.4, SciPy 1.17.1, and statsmodels 0.15.0; the analysis code is available from the corresponding author on request. All tests were two-tailed. Continuous variables are expressed as median and interquartile range. Prior to analysis, we checked the internal arithmetic consistency of the hematology panel by comparing hematocrit to the erythrocyte count multiplied by MCV, MCH to hemoglobin divided by erythrocyte count, and total leukocyte count to the sum of the five absolute differential counts. The main analysis was an ordinary least squares regression of standardized erythrocyte count on standardized FT3, FT4, and log TSH with the inclusion of age and sex as covariates using HC3 heteroskedasticity-consistent standard errors. Effects are reported as standardized beta per 1 standard deviation (SD) with 95% confidence intervals (CIs) and as adjusted differences in native units across the 5th–95th centile of exposure.

We explored non-linearity using restricted cubic splines with four knots at the 5th, 35th, 65th, and 95th centiles and performed a Wald test on the non-linear terms. Stratified estimation was used to assess effect modification by red cell phenotype. Heterogeneity was assessed with the Cochran Q test and quantified with I^2^.

No medication records, inflammatory markers, or reticulocyte counts were available in the export; creatinine and vitamin B12 could be linked for a subset and were used in sensitivity analyses (Section 2.2). To account for a potential contribution of thyroid hormone therapy, we pre-specified a sensitivity analysis excluding records with a biochemical signature of exogenous thyroid hormone, defined as the lowest decile of FT3 to FT4 ratio, or TSH > 4 mIU/L with FT4 ≥ 10 pmol/L, or TSH < 0.4 mIU/L with FT4 > 14.4 pmol/L. E-values were calculated for the primary estimate [20]. Additional sensitivity analyses are reported in Supplementary Table S3. Fewer than 0.2% of records for all primary variables were missing, so a complete-case analysis was performed.

The hypotheses investigated here were generated from an exploratory analysis of this dataset. The analysis plan was locked before the manuscript was written and amended once, when iron indices were linked; it was not registered in a public registry. The sensitivity analyses added during peer review (the same-episode and interval-bounded ferritin subsets, and adjustment for creatinine and vitamin B12) were not pre-specified. As a purely observational study, trial registration was not required. The study is thus hypothesis-generating rather than confirmatory, and all analyses except the primary hypothesis and two pre-specified secondary hypotheses are exploratory. Reporting adheres to the Strengthening the Reporting of Observational Studies in Epidemiology (STROBE) statement [21].

Use of artificial intelligence. Generative artificial intelligence (Claude, Anthropic, San Francisco, CA, USA) was used under the direction of the authors for statistical programming support, figure preparation, and drafting and editing of text; it was not used to generate data or to interpret results. All code, analyses, references, and statements were reviewed and verified by the authors, who take full responsibility for the content. The disclosure statement required by the journal appears once in the Acknowledgments.

## 3. Results

### 3.1. Participants and Cohort Features

**Figure 1:**
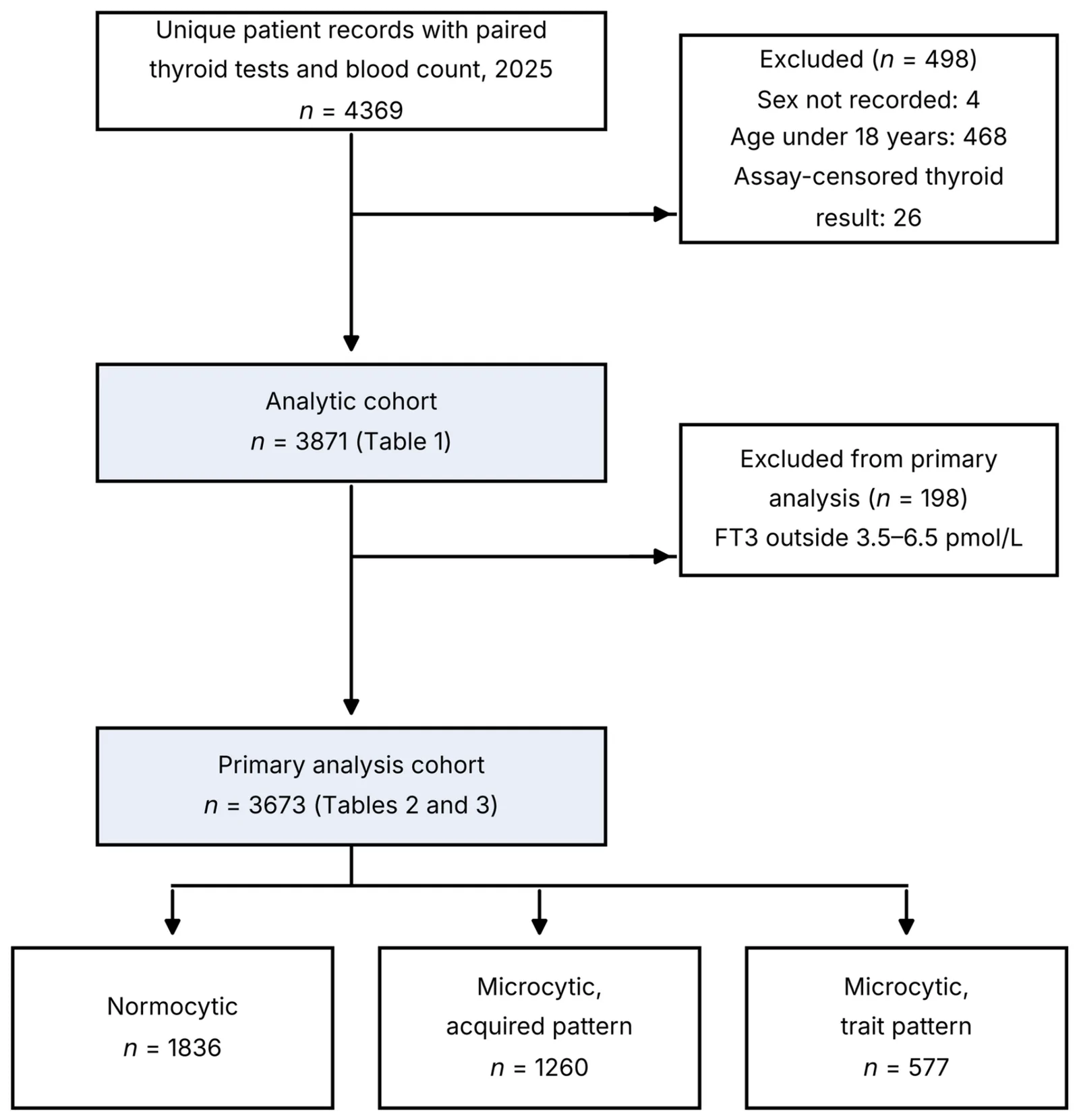
Participant flow. Of 4369 unique patient records, 498 were excluded, leaving an analytic cohort of 3871. The primary analysis was restricted to the 3673 records with free triiodothyronine within the reference interval, which were then stratified by index-defined red cell phenotype (normocytic, microcytic acquired pattern, and microcytic presumed inherited-trait pattern).

Of 4369 unique patient records, four were excluded due to undetermined sex, 468 due to age under 18 years, and 26 due to an assay censoring flag, and 3871 were analyzed. Of these, 3673 had FT3 within the reference interval (Figure 1). The hematology panel had high internal consistency, with hematocrit agreeing with the product of erythrocyte count and MCV (*r* = 0.996), and the total leukocyte count agreeing with the sum of the five absolute differential counts (median absolute difference 0.000). Baseline characteristics are shown in Table 1. The study population was 76.2% female, with a median age of 39 years (interquartile range 30–49 years); 94.0% were Saudi nationals. In 74.0% of the records, the thyroid phenotype was euthyroid. The median MCV was 80.0 fL, 50.0% of records were microcytic, and 45.6% were anemic by World Health Organization (WHO) criteria. In the microcytic records, 15.7% of the cohort met the index definition of the presumed trait pattern and 34.3% met the acquired one. The trait pattern was more common in men (27.4%) than in women (12.1%), while the acquired pattern was the reverse (10.2% and 41.8%) in keeping with menstrual iron loss superimposed on a constitutional background common to both sexes.

### 3.2. Erythrocyte Count and Free Triiodothyronine

After mutual adjustment for FT4 and log TSH and adjustment for age and sex, FT3 was associated with erythrocyte count (standardized beta 0.097, 95% CI 0.066–0.128, *p* < 0.001; model R^2^ 0.218). FT4 was less strongly associated in the same direction (0.053, 0.022–0.084, *p* < 0.001), and log TSH was not associated (0.009, −0.021 to 0.040, *p* = 0.547) (Table 2). In native units, the linear association within the reference interval corresponded to +2.7 g/L of hemoglobin per 1 pmol/L FT3.

Variance inflation factors were 1.06–1.12. FT3 and FT4 were practically uncorrelated (Spearman rho −0.001, *p* = 0.939) and both were weakly correlated with log TSH (−0.084 and −0.139). Excluding the 191 records with Cook’s distance > 4/*n* did not significantly alter the estimate (0.087, *p* < 0.001).

The adjusted difference across the 5th to 95th centile of FT3 (4.0–6.2 pmol/L) was 0.225 × 10^12^/L for erythrocyte count (95% CI 0.150–0.300), 4.76 g/L for hemoglobin (2.83–6.68) and 1.36% for hematocrit (0.810–1.915).

### 3.3. Negative Control Outcomes

Neither FT3, FT4, nor log TSH was associated with MCV (FT3 beta −0.012, 95% CI −0.047 to 0.023, *p* = 0.495) or MCH (−0.003, −0.037 to 0.031, *p* = 0.856) (Table 2). With 3673 records, the smallest detectable correlation at 80.0% power was 0.046, so these are informative null results.

### 3.4. Shape of the Relationship and Effect Modification

**Figure 2:**
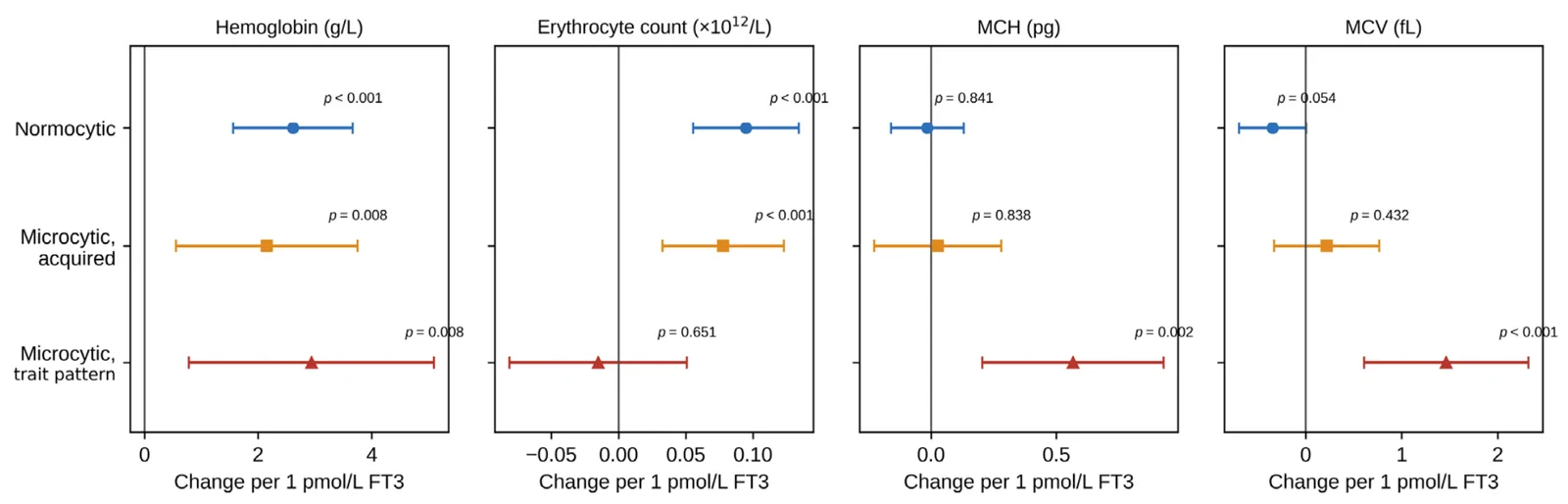
Associations of free triiodothyronine with hemoglobin, erythrocyte count, mean corpuscular hemoglobin, and mean corpuscular volume, stratified by index-defined red cell phenotype. Points are the adjusted change per 1 pmol/L free triiodothyronine with 95% confidence intervals, from models mutually adjusted for free thyroxine and log thyrotropin and adjusted for age and sex, with HC3 robust standard errors. Exact *p* values are shown above each estimate.

**Figure 3:**
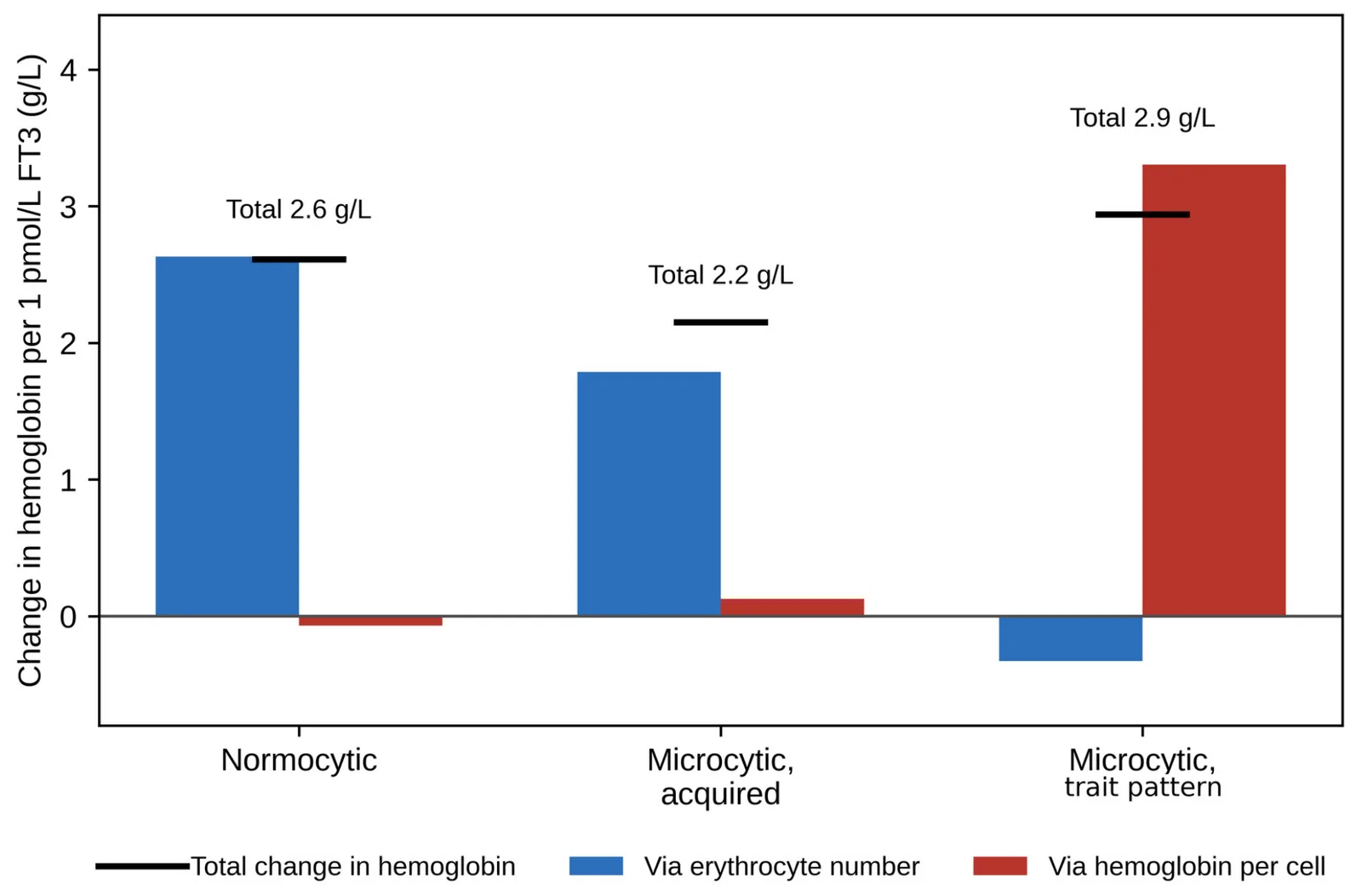
Decomposition of the hemoglobin association by red cell phenotype (hemoglobin = erythrocyte count × mean corpuscular hemoglobin). Bars show the component of the adjusted change in hemoglobin attributable to a change in erythrocyte number and to a change in hemoglobin per cell. Horizontal markers show the total adjusted change in hemoglobin. The total is similar across phenotypes, while its composition is not.

Restricted cubic splines rejected linearity for erythrocyte count (*p* < 0.001), hemoglobin (*p* = 0.002), and hematocrit (*p* < 0.001), but not for MCV (*p* = 0.086). Fitted curves plateaued at ~5.5 pmol/L (Figure S1), with the lower reference interval used as the starting point. No association was observed between log TSH and erythrocyte count in linear or spline models (*p* = 0.135 and *p* = 0.123). When the association was fitted linearly over the whole range of FT3, including values as high as 40.2 pmol/L, the association with hemoglobin was null (0.037, *p* = 0.113), because the thyrotoxic tail, where high FT3 coexists with normal or reduced hemoglobin, flattens the slope. Within the reference interval, the same association was strongly positive (0.085, *p* < 0.001). Stratification by red cell phenotype showed a dissociation between the two erythroid endpoints (Table 3, Figure 2). The FT3–erythrocyte count association was similar in the normocytic records (0.101, 95% CI 0.059–0.144, *p* < 0.001) and acquired microcytic records (0.099, 0.042–0.157, *p* < 0.001), but absent in trait-pattern records (−0.017, −0.093 to 0.058, *p* = 0.651) with significant heterogeneity (Cochran Q = 7.84, *p* = 0.020, I^2^ 74.0%). The FT3–hemoglobin association was different, being similar and significant in all three strata (0.099, *p* < 0.001; 0.079, *p* = 0.008; and 0.089, *p* = 0.008) without heterogeneity (Q = 0.30, *p* = 0.860, I^2^ 0.0%). Decomposing the hemoglobin association into its two components (hemoglobin = erythrocyte count × MCH), the component attributable to erythrocyte number accounted for 101.0% and 83.0% of the total in the normocytic and acquired microcytic strata, respectively, and for none of it in the trait-pattern stratum, where the total was attributable to higher MCH and MCV (Figure 3). Similar conclusions were obtained with unstandardized models. Standard deviations of erythrocyte counts were similar across strata (0.556, 0.447, and 0.508), so variance restriction was not an explanation. Interaction with the trait pattern was significant for erythrocyte count (*p* = 0.002), MCV (*p* = 0.002) and MCH (*p* = 0.021), but not for hemoglobin (*p* = 0.639).

### 3.5. Sensitivity Analysis

Exclusion of the 634 records (17.3%) with a biochemical signature of thyroid hormone therapy attenuated the estimate by about one quarter, but it was still clearly positive (0.074, 95% CI 0.041–0.107, *p* < 0.001). Comparable results were found when the analysis was restricted to strict euthyroid records (0.085, *p* < 0.001) and to outpatients (0.065, *p* < 0.001). This held true after additional adjustment for MCV and red cell distribution width (0.086, *p* < 0.001) and leukocyte count, neutrophil-to-lymphocyte ratio, and platelet count [22] (0.090, *p* < 0.001). Additional adjustment for log creatinine in the 3201 records with a 2025 creatinine result (standardized β 0.090 before and 0.090 after adjustment), for log vitamin B12 in the 2363 records with a B12 result (0.087 and 0.087), and for both in the 2099 records with both (0.081, 95% CI 0.041–0.122) left the estimate essentially unchanged, and the hemoglobin estimates behaved the same way. The full results are reported in Supplementary Table S3.

The association was present in women (0.108, *p* < 0.001) and directionally consistent but not statistically significant in men (0.068, −0.013 to 0.148, *p* = 0.098), and was stronger above age 50 years (0.137, *p* < 0.001) than between 18 and 50 years (0.072, *p* < 0.001). In the single-encounter subgroup, the estimate was attenuated and non-significant (0.036, −0.009 to 0.082, *p* = 0.113). At that sample size, the smallest detectable effect was 0.070, so this is indeterminate rather than a failure to replicate. The E-value was 1.41 for the main estimate and 1.32 for its confidence limit.

Under a stricter trait definition requiring all three discriminant indices to agree (*n* = 317), the FT3–erythrocyte count association remained absent (β 0.011 × 10^12^/L per pmol/L, 95% CI −0.071 to 0.093, *p* = 0.791; interaction with the rest of the cohort *p* = 0.067), while the MCH and hemoglobin estimates in this smaller stratum were positive but not statistically significant (MCH +0.09 pg, *p* = 0.631; hemoglobin +0.9 g/L, *p* = 0.461). The absence of a count association in the trait pattern is therefore robust to the definition used; the hemoglobin-per-cell route suggested by the majority-vote stratum is not and is treated as tentative.

### 3.6. Validation Against Measured Iron Status

Ferritin was available for 1737 patients in the primary cohort and transferrin saturation for 677. Of these, 1711 had a numeric ferritin value and entered the ferritin-adjusted model. For 894 of these (52.3%), ferritin and the index thyroid and blood count tests were drawn in the same laboratory episode; for 782 (45.7%), there were several order dates in 2025, so the interval could not be fixed exactly but could not exceed the span of those dates (median 134 days, interquartile range 62 to 214), and for 98 of these, the span was 30 days or less; for 35 (2.0%), ferritin was available only from 2024, a minimum of 306 days (median; interquartile range 220 to 366) before the index episode. Both ferritin and transferrin saturation differed sharply across the three index-defined strata (Kruskal–Wallis *p* < 0.001 for each; Supplementary Table S4). Median ferritin was 9.2 µg/L in the acquired microcytic stratum, 22.9 µg/L in the trait stratum, and 25.9 µg/L in normocytic records, and measured iron deficiency was present in 65.8%, 40.6%, and 33.3%, respectively. The surrogate classification therefore tracked measured iron status in the direction assumed, although the trait stratum was heterogeneous, as expected of discriminant indices.

FT3 was not associated with any iron index after mutual adjustment (transferrin saturation *p* = 0.282; ferritin *p* = 0.687; iron *p* = 0.071; total iron-binding capacity *p* = 0.141), which argues against an iron-absorption route for the FT3–hemoglobin association. In the ferritin-linked subset, the primary association persisted after adding log ferritin as a covariate (erythrocyte count 0.111 × 10^12^/L per pmol/L FT3, *p* < 0.001; hemoglobin +2.14 g/L per pmol/L FT3, *p* = 0.001). In the same-episode subset (*n* = 894), the FT3 association was 0.094 × 10^12^/L per pmol/L for erythrocyte count (95% CI 0.033 to 0.155, *p* = 0.003) and +2.04 g/L per pmol/L for hemoglobin (95% CI 0.43 to 3.65, *p* = 0.013), and changed little after adjustment for log ferritin (0.093 × 10^12^/L and +1.95 g/L); in the 992 records whose interval could not have exceeded 30 days, the corresponding estimates were 0.102 × 10^12^/L (*p* = 0.001) and +2.54 g/L (*p* = 0.002). These estimates were similar to those in the full linked subset (0.112 × 10^12^/L and +2.30 g/L before adjustment; Supplementary Table S6).

In an exploratory cross-classification by phenotype and measured iron status (Supplementary Table S5), the FT3 association with both erythrocyte count and hemoglobin was strongest in iron-replete normocytic patients (*n* = 662; erythrocyte count β 0.111 × 10^12^/L per pmol/L, *p* = 0.001; hemoglobin +3.6 g/L per pmol/L, *p* < 0.001) and was attenuated and non-significant in iron-deficient normocytic patients (*n* = 331; *p* = 0.429 and 0.395), in iron-deficient acquired microcytic patients (*n* = 482; *p* = 0.234 and 0.485), and in iron-replete trait-pattern patients (*n* = 189; *p* = 0.424 and 0.468). This subset was more often anemic than the remainder of the cohort (51.3% versus 44.0%); the cells are small, and the comparison was not pre-specified.

## 4. Discussion

In 3673 adults from a region where inherited hemoglobin disorders and iron deficiency are both common, FT3, but not TSH, was associated with erythrocyte count and hemoglobin after mutual adjustment, and the association was non-linear, reaching a plateau near 5.5 pmol/L. The central observation is that the erythrocyte count association was conditional on red cell phenotype: it was absent in the index-defined trait pattern, in which erythrocyte count is already high, under both a majority-vote and a stricter definition of that pattern, whereas the hemoglobin association did not differ by phenotype. In an exploratory analysis, both associations were also blunted in measured iron deficiency. The clearest association was seen where neither constraint applied, in iron-replete normocytic patients, in whom each 1 pmol/L of FT3 was associated with 3.6 g/L more hemoglobin. We interpret this pattern as consistent with a limit on the marrow’s count response, but the design cannot establish that mechanism, and the alternatives are set out below.

A ceiling on the count response is one interpretation of the trait-pattern result, and it is a hypothesis rather than a finding. Bone marrow activity was not assessed: reticulocyte count, erythropoietin, and marrow examination were unavailable, so the data describe the association of FT3 with circulating cell number, not with erythropoietic output. What the data do show is that in the trait stratum the median erythrocyte count was 5.73 × 10^12^/L and 73.0% of records exceeded 5.5 × 10^12^/L, against 9.0% of normocytic records. Thalassemia trait is characterized by a compensatory increase in erythrocyte number driven by globin chain imbalance, and a marrow already near that limit would have little room to respond to a further proliferative stimulus. Variance restriction was excluded as an explanation: the standard deviation of erythrocyte count in trait-pattern records (0.508 × 10^12^/L) was comparable to that in normocytic records (0.556), and unstandardized models gave the same result. Misclassification is the other explanation that must be considered. The trait pattern was defined by discriminant indices, which misclassify a meaningful proportion of cases [19], and the stratum will contain some iron-deficient records with a preserved count as well as miss some carriers whose MCV is normal. Non-differential misclassification of an effect modifier generally attenuates the true difference between strata [23], so the observed interaction is more likely an underestimate than an artifact. Misclassification that is differential with respect to FT3 is the more serious possibility: the indices are computed from MCV, erythrocyte count and hemoglobin, which are also the outcomes, so if thyroid status moved these values enough to shift records across an index cut-off, the stratum contrast could be inflated. The null FT3 associations with MCV and MCH in the primary analysis make large shifts of this kind unlikely, but the possibility cannot be excluded with the available data, which is why the main conclusion is framed around the observed laboratory phenotype rather than a genetically confirmed category.

Erythrocyte count and hemoglobin are related but not interchangeable outcomes, and they behaved differently here. Hemoglobin concentration is the product of erythrocyte number and the hemoglobin content of each cell, so an FT3 association with hemoglobin can arise through either term. In the normocytic and acquired microcytic strata, the hemoglobin association was carried almost entirely by erythrocyte number, with no contribution from MCH; in the trait stratum, the count association was absent, and a hemoglobin association of similar size was carried by MCH and MCV. The primary result is therefore a phenotype-dependent association with cell number, which the hemoglobin result supports in two strata and diverges from in the third. We do not treat the count association as direct evidence of increased erythropoietic activity, which would require reticulocyte or marrow data; an association with circulating cell number is also compatible with altered red cell survival or plasma volume, both of which thyroid hormone influences.

The majority-vote trait stratum suggested that the hemoglobin increment might be preserved there through higher MCH and MCV rather than through count. We report this but treat it as tentative, because it did not reach significance under the stricter trait definition or in iron-replete trait-pattern records, both of which are small. A specific mechanism would be available if it were confirmed: thyroid hormone is required for terminal erythroid differentiation through thyroid hormone receptor beta acting with NCOA4 [3], and NCOA4 is the ferritinophagy receptor that supplies iron for heme synthesis, so the same pathway that drives terminal maturation governs hemoglobinization. This is a hypothesis for a genotyped cohort to test.

The linked iron data bear on the alternative explanation that thyroid hormone acts through iron availability. FT3 showed no association with transferrin saturation, ferritin, serum iron, or total iron-binding capacity, and the primary association persisted after adjustment for ferritin and when restricted to records in which ferritin and the index tests were drawn in the same episode. The iron-absorption route is therefore not supported by direct measurement. What iron status did do was modify the response: where hemoglobinization was iron-limited, neither the count nor the hemoglobin association was clearly present. That is what a marrow-level effect that requires substrate would look like, and it is consistent with the trait-pattern result in showing that the response is expressed only where capacity exists. Because iron studies were requested selectively and the cross-classified groups are small, this is reported as exploratory.

The regulation of iron supply to the erythron offers a framework for these observations. Hepcidin, the hepatic hormone that controls plasma iron, binds the iron exporter ferroportin on enterocytes and macrophages and induces its degradation, which reduces iron absorption and release from stores [24,25]. Erythroferrone, produced by erythroblasts in response to erythropoietin, suppresses hepcidin and so increases iron availability when erythropoiesis expands [26,27]. Thalassemia syndromes with ineffective erythropoiesis overproduce erythroferrone and suppress hepcidin [27], which may contribute to the higher ferritin we observed in the trait stratum than in the acquired microcytic stratum, although carrier states perturb this axis far less than disease. Whether thyroid hormone acts on this axis in humans is unsettled: hepcidin fell after restoration of euthyroidism in hypothyroid patients and correlated with FT3 before treatment [28], whereas treatment of hyperthyroidism did not change hepcidin-25 [29]. Our data do not resolve this, because hepcidin, erythroferrone, and soluble ferroportin were not measured. The absence of any FT3 association with ferritin, iron, or transferrin saturation argues against a large effect on systemic iron supply within the reference interval, but a modest effect on iron delivery to the erythroblast, which these indices would not capture, remains possible. Measuring this regulatory panel alongside thyroid function, as has been done in other clinical cohorts [30], would allow the iron-supply and marrow-capacity explanations to be separated.

That FT3, rather than TSH, carries the erythroid signal is consistent with Mendelian randomization evidence that deiodinase-regulated thyroid hormone influences erythropoiesis in euthyroid individuals, while euthyroid TSH does not [9]. The present study adds that the association is conditional on red cell phenotype and, tentatively, on iron status, which a population with a low prevalence of inherited microcytosis could not have detected. These results may also help explain why the link between thyroid function and anemia has remained ambiguous [5]: if the signal is carried by FT3, and its expression depends on red cell phenotype and iron status, studies using TSH alone in populations of mixed iron status would be expected to give weak and unstable associations. The hypothalamic–pituitary–thyroid set-point is itself not fixed [31], which further limits TSH as a proxy for tissue thyroid hormone action. A further methodological caution for that literature is that linear modeling across the full measured range of FT3, including thyrotoxic values, produced a false null (β 0.037, *p* = 0.113), whereas the same association within the reference interval was strongly positive. A curved FT3 relationship was also the best fit in the Busselton cohort [7].

The principal strength is the population itself. The high prevalence of both constitutional and acquired microcytosis provides a natural experiment in which erythrocyte production is constrained in two mechanistically opposite ways, and the conditional association could not have been observed in a homogeneous red cell background. All three analytes were measured in every participant alongside a complete blood count from the same laboratory episode; the sample is large enough that the TSH null results are informative (smallest detectable correlation 0.046) rather than underpowered; the negative control outcomes [15] were null in the primary analysis; and the phenotype classification was consistent with measured iron status in the expected direction.

The limitations fall under five headings. First, the design is cross-sectional, the indication for testing is unknown, and selection into the cohort may depend partly on the outcome, a collider structure that adjustment cannot repair; no direction of effect can be inferred. Second, genetic confirmation was incomplete. The trait pattern rests on discriminant indices that misclassify a meaningful proportion of cases [19], and hemoglobin electrophoresis was available for too few patients to validate it. We classified 15.7% of records as the trait pattern (27.4% of men), against 4.4% with suspected alpha thalassemia in premarital screening in the same region [10]. The two figures are not directly comparable. The 4.4% refers to alpha thalassemia alone, and the same screening program found sickle cell trait in 13.5%, beta thalassemia trait in 1.5%, and beta thalassemia with sickle cell trait in 2.5% [32], any of which can produce the index pattern when microcytic; electrophoresis does not detect most alpha thalassemia; the premarital population is young and self-selected, whereas ours was a hospital population with a 45.6% prevalence of anemia; and among healthy male university students in Jazan, 37.6% had a low MCV with a high erythrocyte count suggestive of a carrier state [33]. The index-based estimate is nevertheless likely to over-identify the trait pattern, and the consequences of that misclassification are discussed above. Third, iron testing was selective and, for about half of the linked records, not demonstrably contemporaneous with the index episode, although the estimates in the same-episode subset were similar; the linked subset was more often anemic than the remainder, the cross-classified groups were small, and the iron-status comparison was not pre-specified. Fourth, confounding is incompletely addressed. Medication records were unavailable, so thyroid hormone replacement and antithyroid therapy could not be identified directly; excluding records with a biochemical signature of exogenous thyroid hormone attenuated the estimate by about one quarter without abolishing it, but residual misclassification is certain. Renal function and vitamin B12 could be examined only in subsets, where adjustment for creatinine and vitamin B12 did not change the estimate; inflammatory markers, folate, and reticulocyte counts were unavailable or too sparse, so acute or chronic inflammation with non-thyroidal illness [34,35] and folate deficiency cannot be excluded as common causes of lower FT3 and altered red cell indices, although FT3 was unrelated to ferritin, an acute-phase reactant, and additional adjustment for leukocyte count and neutrophil-to-lymphocyte ratio did not change the estimate. The E-value of 1.41 indicates that unmeasured confounding of moderate strength could account for the primary finding. Fifth, generalizability is limited. The cohort was 76.2% female with pregnancy status unrecorded; the male subgroup was underpowered (β 0.068, *p* = 0.098) and no sex-specific inference is drawn; samples were collected across the full 24 h, so diurnal variation in TSH is unmodeled; the findings apply to the FT3 reference interval in one region; and the hypotheses were generated by exploratory analysis of this same dataset, with the plan locked before writing and amended openly when the iron data were linked.

These findings have no immediate diagnostic application. Thyroid function explained 0.4% of the variance in erythrocyte count over and above age and sex, a population-level signal without individual predictive value. The interest is mechanistic and methodological. If the erythroid association with thyroid hormone depends on red cell phenotype, then studies of thyroid function and anemia conducted in populations that differ in hemoglobinopathy prevalence or iron status should not be expected to agree, and reporting erythrocyte number and erythrocyte indices separately, with iron status, would make such studies comparable. Replication in a cohort with confirmed hemoglobin genotype, measured iron status in all participants, reticulocyte count or erythropoietin, and documented medication history is the necessary next step.

## 5. Conclusions

In 3673 adults from a region where inherited hemoglobin disorders are common, free triiodothyronine rather than thyrotropin was associated with erythrocyte count and hemoglobin. The erythrocyte count association was absent in an index-defined, presumed inherited-trait pattern in which erythrocyte number was already high, under two definitions of that phenotype, and in exploratory analysis, both associations were blunted where hemoglobinization was iron-limited. Linked iron indices were consistent with the phenotype classification and showed no association between free triiodothyronine and iron status. These observations are compatible with a limit on the marrow’s count response but do not demonstrate one, because erythropoietic activity was not measured. They are hypothesis-generating and require replication in a cohort with confirmed hemoglobin genotype, measured iron status throughout, and reticulocyte or erythropoietin data.

## Figures and Tables

**Table 1:** Baseline characteristics of the analytic cohort, by sex.

Characteristic	All (*n* = 3871)	Women (*n* = 2951)	Men (*n* = 920)	*p*
Age, years	39.00 (30.00 to 49.00)	38.00 (29.00 to 48.00)	42.00 (32.00 to 53.00)	<0.001
Saudi nationality, *n* (%)	3640 (94.0)	2805 (95.1)	835 (90.8)	<0.001
Outpatient encounters only, *n* (%)	2842 (73.4)	2169 (73.5)	673 (73.2)	0.868
Thyroid function				
FT3, pmol/L	5.02 (4.59 to 5.46)	4.95 (4.54 to 5.37)	5.24 (4.80 to 5.69)	<0.001
FT4, pmol/L	9.80 (8.57 to 11.16)	9.72 (8.52 to 11.11)	10.00 (8.74 to 11.31)	0.008
TSH, mIU/L	2.36 (1.44 to 3.82)	2.50 (1.48 to 4.12)	1.98 (1.30 to 3.07)	<0.001
FT3 to FT4 ratio	0.51 (0.44 to 0.60)	0.51 (0.43 to 0.59)	0.53 (0.45 to 0.62)	<0.001
Thyroid phenotype, *n* (%)				<0.001
Euthyroid	2863 (74.0)	2098 (71.1)	765 (83.2)	
Subclinical hypothyroid	725 (18.7)	613 (20.8)	112 (12.2)	
Overt hypothyroid	178 (4.6)	152 (5.2)	26 (2.8)	
Subclinical hyperthyroid	53 (1.4)	45 (1.5)	8 (0.9)	
Overt hyperthyroid	52 (1.3)	43 (1.5)	9 (1.0)	
Red cell indices				
RBC, ×10^12^/L	4.84 (4.46 to 5.31)	4.69 (4.36 to 5.07)	5.42 (5.01 to 5.79)	<0.001
Hemoglobin, g/L	122 (111 to 134)	118 (108 to 126)	144 (135 to 152)	<0.001
Hematocrit, %	38.30 (35.10 to 41.70)	37.10 (34.40 to 39.60)	44.10 (41.60 to 46.48)	<0.001
MCV, fL	79.99 (73.37 to 84.83)	79.10 (72.30 to 84.30)	82.08 (77.07 to 86.40)	<0.001
MCH, pg	25.64 (22.80 to 27.60)	25.20 (22.40 to 27.25)	27.00 (24.88 to 28.70)	<0.001
MCHC, g/L	319 (309 to 329)	317 (307 to 326)	326 (316 to 336)	<0.001
RDW, %	14.00 (13.00 to 15.60)	14.30 (13.20 to 15.90)	13.30 (12.40 to 14.30)	<0.001
Anemia (WHO), *n* (%)	1767 (45.6)	1628 (55.2)	139 (15.1)	<0.001
Microcytosis (MCV < 80 fL), *n* (%)	1936 (50.0)	1590 (53.9)	346 (37.6)	<0.001
Red cell phenotype, *n* (%)				<0.001
Normocytic	1935 (50.0)	1361 (46.1)	574 (62.4)	
Microcytic, acquired pattern	1327 (34.3)	1233 (41.8)	94 (10.2)	
Microcytic, trait pattern	609 (15.7)	357 (12.1)	252 (27.4)	
Leukocytes and platelets				
WBC, ×10^9^/L	6.22 (5.07 to 7.58)	6.34 (5.19 to 7.65)	5.70 (4.80 to 7.20)	<0.001
Neutrophils, ×10^9^/L	2.62 (1.93 to 3.59)	2.67 (1.99 to 3.64)	2.43 (1.79 to 3.42)	<0.001
Lymphocytes, ×10^9^/L	2.70 (2.16 to 3.29)	2.76 (2.21 to 3.38)	2.50 (2.00 to 3.01)	<0.001
Neutrophils, %	43.10 (35.80 to 51.00)	43.21 (36.10 to 51.05)	42.60 (34.76 to 50.90)	0.302
Lymphocytes, %	44.10 (36.60 to 50.80)	44.20 (36.70 to 50.90)	43.40 (36.27 to 50.42)	0.055
Platelets, ×10^9^/L	316.00 (261.00 to 376.00)	330.00 (275.00 to 391.00)	272.00 (228.00 to 325.00)	<0.001
MPV, fL	10.60 (9.90 to 11.40)	10.70 (10.00 to 11.50)	10.40 (9.80 to 11.10)	<0.001

Data are median (interquartile range) unless indicated otherwise. Anemia by WHO thresholds (hemoglobin below 120 g/L in women and 130 g/L in men). Red cell phenotype assigned among microcytic records by majority vote of the Mentzer, Green and King, and England and Fraser indices; “trait pattern” denotes a presumed inherited-trait pattern defined by these indices, not a confirmed hemoglobinopathy. FT3, free triiodothyronine; FT4, free thyroxine; TSH, thyrotropin; MCV, mean corpuscular volume; MCH, mean corpuscular hemoglobin; MCHC, mean corpuscular hemoglobin concentration; RDW, red cell distribution width; MPV, mean platelet volume; RBC, red blood cell (erythrocyte) count; WBC, white blood cell (leukocyte) count; WHO, World Health Organization.

**Table 2:** Mutually adjusted associations of thyroid analytes with erythroid indices.

Outcome	Hypothesis	*n*	Analyte	Standardized β (95% CI)	*p*
Erythrocyte count	H1 primary	3673	FT3	+0.097 (+0.066 to +0.128)	<0.001
			FT4	+0.053 (+0.022 to +0.084)	<0.001
			log TSH	+0.009 (−0.021 to +0.040)	0.547
Mean corpuscular volume	H2 negative control	3673	FT3	−0.012 (−0.047 to +0.023)	0.495
			FT4	−0.012 (−0.044 to +0.021)	0.478
			log TSH	+0.006 (−0.026 to +0.038)	0.725
Mean corpuscular hemoglobin	H2 negative control	3673	FT3	−0.003 (−0.037 to +0.031)	0.856
			FT4	−0.008 (−0.039 to +0.024)	0.640
			log TSH	−0.005 (−0.036 to +0.026)	0.751
Hemoglobin	secondary	3673	FT3	+0.085 (+0.056 to +0.114)	<0.001
			FT4	+0.039 (+0.010 to +0.067)	0.008
			log TSH	+0.005 (−0.022 to +0.032)	0.731
Hematocrit	secondary	3673	FT3	+0.091 (+0.061 to +0.120)	<0.001
			FT4	+0.042 (+0.012 to +0.071)	0.006
			log TSH	+0.014 (−0.013 to +0.042)	0.312

Ordinary least squares regression with HC3 robust standard errors; FT3, FT4, and log TSH entered simultaneously; adjusted for age and sex; restricted to FT3 within 3.5–6.5 pmol/L. MCV and MCH were pre-specified negative control outcomes. H1, primary hypothesis; H2, pre-specified negative control hypothesis; FT3, free triiodothyronine; FT4, free thyroxine; TSH, thyrotropin; MCV, mean corpuscular volume; MCH, mean corpuscular hemoglobin; CI, confidence interval; HC3, heteroskedasticity-consistent covariance estimator, type 3.

**Table 3:** Association of free triiodothyronine with erythrocyte count and hemoglobin, by red cell phenotype.

Outcome	Red Cell Phenotype	*n*	FT3 β (95% CI)	*p*
Erythrocyte count	Normocytic	1836	+0.101 (+0.059 to +0.144)	<0.001
	Microcytic, acquired pattern	1260	+0.099 (+0.042 to +0.157)	<0.001
	Microcytic, trait pattern	577	−0.017 (−0.093 to +0.058)	0.651
	Heterogeneity		Q = 7.84, I^2^ = 74%	0.020
Hemoglobin	Normocytic	1836	+0.099 (+0.059 to +0.138)	<0.001
	Microcytic, acquired pattern	1260	+0.079 (+0.020 to +0.138)	0.008
	Microcytic, trait pattern	577	+0.089 (+0.024 to +0.155)	0.008
	Heterogeneity		Q = 0.30, I^2^ = 0%	0.860

Standardized β per one standard deviation of FT3, mutually adjusted for FT4 and log TSH and adjusted for age and sex. Heterogeneity tested by Cochran Q (2 degrees of freedom). FT3, free triiodothyronine; FT4, free thyroxine; TSH, thyrotropin; CI, confidence interval.

## Data Availability

Restrictions apply to the availability of these data. Data were obtained from the Jazan Health Cluster (Baish General Hospital, King Fahad Hospital, and the Endocrinology and Diabetes Center) as routine patient laboratory records containing potentially re-identifiable information and are available from the authors with the permission of the Jazan Health Cluster.

## Supplementary Materials

The following supporting information can be downloaded at: https://www.mdpi.com/article/10.3390/diagnostics16213587/s1. Table S1: Correlations of thyroid analytes with erythroid and platelet indices; Table S2: Tests of departure from linearity; Table S3. Sensitivity analyses for the primary association; Table S3 (addendum): Sensitivity analyses with additional adjustment for serum creatinine and vitamin B12, standardized β per 1 SD of FT3; Table S4: Iron indices and measured iron deficiency by red cell phenotype (primary cohort, linked subset); Table S5: FT3 associations cross-classified by red cell phenotype and measured iron status (exploratory); Table S6: Association of free triiodothyronine with erythrocyte count and hemoglobin in the full ferritin-linked subset, the same-episode subset and the interval-bounded subset, with and without adjustment for log ferritin; Figure S1: Adjusted dose–response curves by red cell phenotype.
