# Supplementary material for: Red Cell Phenotype Modifies the Association Between Free Triiodothyronine and Erythrocyte Count: A Cross-Sectional Study of 3673 Adults in a Hemoglobinopathy-Endemic Region of Saudi Arabia
Source: Diagnostics (Basel). 2026 Oct 30;16(21):3587. doi: 10.3390/diagnostics16213587 (PMC13761865; doi:10.3390/diagnostics16213587)
Supplement: Supplementary file 1 [file diagnostics-16-03587-s001.zip › diagnostics-4637892-supplementary.pdf]

Supplementary Materials

Red Cell Phenotype Modifies the Association Between Free Triiodothyronine and Erythrocyte Count: A Cross-Sectional Study of 3673 Adults in a Hemoglobinopathy-Endemic Region of Saudi Arabia

Yahia A. Mjery, Saif Elden B. Abdalla, Tagwa Y. Elsayed, Edrous Alamer, Hazem Mathkour, Abdullah Farasani, Ali Baity, Fahd Alaajam, Faisal Madkhali and Nahla Kambal

Contents

Table S1. Correlations of thyroid analytes with erythroid and platelet indices

Table S2. Tests of departure from linearity

Table S3. Sensitivity analyses for the primary association

Table S3 (addendum). Sensitivity analyses with additional adjustment for serum creatinine and vitamin B12, standardized β per 1 SD of FT3

Table S4. Iron indices and measured iron deficiency by red cell phenotype (primary cohort, linked subset)

Table S5. FT3 associations cross-classified by red cell phenotype and measured iron status (exploratory)

Table S6. Association of free triiodothyronine with erythrocyte count and hemoglobin in the full ferritin-linked subset, the same-episode subset and the interval-bounded subset, with and without adjustment for log ferritin

Figure S1. Adjusted dose–response curves by red cell phenotype

Table S1. Correlations of thyroid analytes with erythroid and platelet indices

Cohort	Thyroid analyte	Blood count variable	N	Crude rho	Adjusted rho	95% CI lower	95% CI upper	P	q (FDR)
Full cohort	lnTSH	RBC	3871	-0.051	0.011	-0.020	0.043	0.486	0.629
Full cohort	lnTSH	Hemoglobin	3871	-0.075	-0.007	-0.038	0.025	0.672	0.788
Full cohort	lnTSH	Hematocrit	3871	-0.058	0.012	-0.020	0.043	0.466	0.618
Full cohort	lnTSH	MCV	3871	-0.012	-0.003	-0.034	0.029	0.873	0.931
Full cohort	lnTSH	MCH	3871	-0.037	-0.019	-0.050	0.013	0.246	0.369
Full cohort	lnTSH	MCHC	3871	-0.083	-0.056	-0.087	-0.024	0.001	0.003
Full cohort	lnTSH	RDW	3871	0.066	0.040	0.008	0.071	0.013	0.042
Full cohort	lnTSH	Platelets	3871	0.008	0.001	-0.030	0.033	0.936	0.962
Full cohort	lnTSH	MPV	3715	0.037	0.028	-0.004	0.060	0.083	0.158
Full cohort	FT4	RBC	3871	0.084	0.073	0.042	0.104	<0.001	<0.001
Full cohort	FT4	Hemoglobin	3871	0.062	0.046	0.015	0.078	0.004	0.016
Full cohort	FT4	Hematocrit	3871	0.069	0.054	0.023	0.086	0.001	0.004
Full cohort	FT4	MCV	3871	-0.014	-0.021	-0.053	0.010	0.185	0.301
Full cohort	FT4	MCH	3871	-0.002	-0.012	-0.043	0.020	0.463	0.618
Full cohort	FT4	MCHC	3871	0.022	0.011	-0.021	0.043	0.496	0.630
Full cohort	FT4	RDW	3871	-0.027	-0.017	-0.048	0.015	0.295	0.425
Full cohort	FT4	Platelets	3871	0.004	0.017	-0.015	0.048	0.299	0.428
Full cohort	FT4	MPV	3715	-0.036	-0.032	-0.064	0.001	0.055	0.116
Full cohort	FT3	RBC	3871	0.194	0.114	0.083	0.145	<0.001	<0.001
Full cohort	FT3	Hemoglobin	3871	0.178	0.099	0.068	0.130	<0.001	<0.001
Full cohort	FT3	Hematocrit	3871	0.185	0.110	0.079	0.141	<0.001	<0.001
Full cohort	FT3	MCV	3871	-0.015	-0.023	-0.054	0.009	0.157	0.265
Full cohort	FT3	MCH	3871	0.007	-0.016	-0.047	0.016	0.321	0.450
Full cohort	FT3	MCHC	3871	0.043	-0.001	-0.032	0.031	0.967	0.967
Full cohort	FT3	RDW	3871	-0.079	-0.043	-0.074	-0.011	0.007	0.027
Full cohort	FT3	Platelets	3871	0.078	0.075	0.044	0.107	<0.001	<0.001
Full cohort	FT3	MPV	3715	-0.008	0.003	-0.030	0.035	0.881	0.931
Full cohort	FT3_FT4	RBC	3871	0.041	0.002	-0.030	0.033	0.917	0.962
Full cohort	FT3_FT4	Hemoglobin	3871	0.049	0.013	-0.018	0.045	0.413	0.574
Full cohort	FT3_FT4	Hematocrit	3871	0.047	0.011	-0.020	0.043	0.480	0.626
Full cohort	FT3_FT4	MCV	3871	0.000	0.001	-0.030	0.033	0.950	0.967
Full cohort	FT3_FT4	MCH	3871	0.005	-0.001	-0.033	0.030	0.936	0.962
Full cohort	FT3_FT4	MCHC	3871	0.008	-0.008	-0.040	0.024	0.617	0.746
Full cohort	FT3_FT4	RDW	3871	-0.023	-0.009	-0.040	0.023	0.594	0.731
Full cohort	FT3_FT4	Platelets	3871	0.040	0.030	-0.002	0.061	0.062	0.129
Full cohort	FT3_FT4	MPV	3715	0.015	0.019	-0.013	0.051	0.256	0.380
Full cohort	TSHI	RBC	3871	-0.021	0.036	0.004	0.067	0.027	0.074
Full cohort	TSHI	Hemoglobin	3871	-0.050	0.009	-0.022	0.041	0.553	0.691
Full cohort	TSHI	Hematocrit	3871	-0.030	0.032	0.001	0.064	0.044	0.102
Full cohort	TSHI	MCV	3871	-0.011	-0.005	-0.037	0.026	0.736	0.841
Full cohort	TSHI	MCH	3871	-0.034	-0.021	-0.052	0.011	0.199	0.315
Full cohort	TSHI	MCHC	3871	-0.076	-0.054	-0.086	-0.023	0.001	0.004
Full cohort	TSHI	RDW	3871	0.054	0.033	0.001	0.064	0.041	0.102
Full cohort	TSHI	Platelets	3871	0.004	0.004	-0.028	0.036	0.801	0.890
Full cohort	TSHI	MPV	3715	0.023	0.016	-0.016	0.049	0.318	0.450
Full cohort	TFQI	RBC	3871	0.029	0.067	0.036	0.098	<0.001	<0.001
Full cohort	TFQI	Hemoglobin	3871	-0.006	0.033	0.001	0.064	0.043	0.102
Full cohort	TFQI	Hematocrit	3871	0.013	0.054	0.022	0.085	0.001	0.004
Full cohort	TFQI	MCV	3871	-0.020	-0.019	-0.051	0.012	0.225	0.342
Full cohort	TFQI	MCH	3871	-0.031	-0.025	-0.057	0.006	0.116	0.207
Full cohort	TFQI	MCHC	3871	-0.047	-0.036	-0.067	-0.004	0.026	0.072
Full cohort	TFQI	RDW	3871	0.029	0.017	-0.015	0.049	0.290	0.423
Full cohort	TFQI	Platelets	3871	0.005	0.011	-0.021	0.043	0.495	0.630
Full cohort	TFQI	MPV	3715	0.004	0.001	-0.031	0.033	0.961	0.967
Euthyroid only	lnTSH	RBC	2863	-0.051	-0.009	-0.046	0.027	0.610	0.756
Euthyroid only	lnTSH	Hemoglobin	2863	-0.068	-0.023	-0.060	0.013	0.213	0.391
Euthyroid only	lnTSH	Hematocrit	2863	-0.061	-0.016	-0.053	0.021	0.392	0.571
Euthyroid only	lnTSH	MCV	2863	-0.022	-0.015	-0.051	0.022	0.425	0.608
Euthyroid only	lnTSH	MCH	2863	-0.040	-0.026	-0.062	0.011	0.172	0.344
Euthyroid only	lnTSH	MCHC	2863	-0.055	-0.035	-0.072	0.001	0.060	0.176
Euthyroid only	lnTSH	RDW	2863	0.056	0.036	-0.000	0.073	0.053	0.165
Euthyroid only	lnTSH	Platelets	2863	0.005	0.002	-0.035	0.038	0.934	0.974
Euthyroid only	lnTSH	MPV	2747	0.040	0.035	-0.003	0.072	0.069	0.180
Euthyroid only	FT4	RBC	2863	0.104	0.099	0.062	0.135	<0.001	<0.001

Cohort	Thyroid analyte	Blood count variable	N	Crude rho	Adjusted rho	95% CI lower	95% CI upper	P	q (FDR)
Euthyroid only	FT4	Hemoglobin	2863	0.075	0.065	0.029	0.102	0.001	0.005
Euthyroid only	FT4	Hematocrit	2863	0.091	0.085	0.049	0.121	<0.001	<0.001
Euthyroid only	FT4	MCV	2863	-0.003	-0.010	-0.047	0.026	0.578	0.747
Euthyroid only	FT4	MCH	2863	-0.001	-0.012	-0.049	0.025	0.515	0.690
Euthyroid only	FT4	MCHC	2863	0.004	-0.007	-0.044	0.029	0.694	0.812
Euthyroid only	FT4	RDW	2863	-0.016	-0.006	-0.042	0.031	0.757	0.856
Euthyroid only	FT4	Platelets	2863	-0.004	0.010	-0.026	0.047	0.579	0.747
Euthyroid only	FT4	MPV	2747	-0.033	-0.029	-0.066	0.009	0.135	0.302
Euthyroid only	FT3	RBC	2863	0.205	0.112	0.076	0.148	<0.001	<0.001
Euthyroid only	FT3	Hemoglobin	2863	0.210	0.107	0.071	0.143	<0.001	<0.001
Euthyroid only	FT3	Hematocrit	2863	0.215	0.119	0.083	0.155	<0.001	<0.001
Euthyroid only	FT3	MCV	2863	0.007	-0.012	-0.049	0.025	0.526	0.698
Euthyroid only	FT3	MCH	2863	0.031	-0.007	-0.044	0.029	0.698	0.812
Euthyroid only	FT3	MCHC	2863	0.056	0.000	-0.037	0.037	0.999	0.999
Euthyroid only	FT3	RDW	2863	-0.092	-0.040	-0.077	-0.004	0.032	0.115
Euthyroid only	FT3	Platelets	2863	0.073	0.086	0.050	0.122	<0.001	<0.001
Euthyroid only	FT3	MPV	2747	-0.014	-0.000	-0.038	0.037	0.992	0.999
Euthyroid only	FT3_FT4	RBC	2863	0.033	-0.017	-0.054	0.019	0.358	0.548
Euthyroid only	FT3_FT4	Hemoglobin	2863	0.057	0.005	-0.032	0.042	0.795	0.877
Euthyroid only	FT3_FT4	Hematocrit	2863	0.049	-0.004	-0.040	0.033	0.845	0.911
Euthyroid only	FT3_FT4	MCV	2863	0.004	-0.001	-0.037	0.036	0.967	0.983
Euthyroid only	FT3_FT4	MCH	2863	0.017	0.004	-0.033	0.041	0.823	0.901
Euthyroid only	FT3_FT4	MCHC	2863	0.028	0.005	-0.031	0.042	0.777	0.864
Euthyroid only	FT3_FT4	RDW	2863	-0.039	-0.017	-0.053	0.020	0.369	0.560
Euthyroid only	FT3_FT4	Platelets	2863	0.043	0.038	0.002	0.075	0.041	0.135
Euthyroid only	FT3_FT4	MPV	2747	0.014	0.019	-0.018	0.056	0.321	0.507
Euthyroid only	TSHI	RBC	2863	-0.000	0.034	-0.003	0.071	0.068	0.180
Euthyroid only	TSHI	Hemoglobin	2863	-0.027	0.009	-0.028	0.045	0.635	0.775
Euthyroid only	TSHI	Hematocrit	2863	-0.013	0.025	-0.012	0.061	0.183	0.356
Euthyroid only	TSHI	MCV	2863	-0.017	-0.015	-0.051	0.022	0.425	0.608
Euthyroid only	TSHI	MCH	2863	-0.033	-0.026	-0.063	0.011	0.162	0.330
Euthyroid only	TSHI	MCHC	2863	-0.048	-0.036	-0.072	0.001	0.057	0.173
Euthyroid only	TSHI	RDW	2863	0.039	0.026	-0.011	0.063	0.163	0.330
Euthyroid only	TSHI	Platelets	2863	-0.001	0.005	-0.031	0.042	0.768	0.859
Euthyroid only	TSHI	MPV	2747	0.022	0.019	-0.018	0.057	0.307	0.495
Euthyroid only	TFQI	RBC	2863	0.057	0.076	0.040	0.113	<0.001	0.001
Euthyroid only	TFQI	Hemoglobin	2863	0.025	0.043	0.007	0.080	0.020	0.088
Euthyroid only	TFQI	Hematocrit	2863	0.042	0.064	0.027	0.100	0.001	0.006
Euthyroid only	TFQI	MCV	2863	-0.014	-0.016	-0.053	0.020	0.380	0.562
Euthyroid only	TFQI	MCH	2863	-0.023	-0.024	-0.061	0.013	0.197	0.375
Euthyroid only	TFQI	MCHC	2863	-0.028	-0.026	-0.063	0.011	0.162	0.330
Euthyroid only	TFQI	RDW	2863	0.017	0.014	-0.022	0.051	0.444	0.622
Euthyroid only	TFQI	Platelets	2863	-0.002	0.010	-0.027	0.047	0.595	0.756
Euthyroid only	TFQI	MPV	2747	-0.002	-0.001	-0.039	0.036	0.948	0.974

Partial Spearman correlations adjusted for age and sex. q values are Benjamini-Hochberg false discovery rate adjusted within each cohort. Leukocyte outcomes are omitted: the association of thyrotropin with the leukocyte differential is the subject of a separate report and is not presented here. Abbreviations: CI, confidence interval; FDR, false discovery rate.

Table S2. Tests of departure from linearity

Exposure	Outcome	P (overall)	P (non-linearity)
FT3	RBC	<0.001	<0.001
FT3	Hemoglobin	<0.001	<0.001
FT3	Hematocrit	<0.001	<0.001
FT3	MCV	0.330	0.489
FT3	MCH	0.142	0.186
FT3	RDW	0.064	0.029
FT3	Platelets	0.002	0.001
FT3	MPV	0.586	0.425
FT3	WBC	0.002	<0.001
FT3	NEU#	0.003	0.003
FT3	LYM#	<0.001	<0.001
FT3	MON#	<0.001	0.066
FT3	NLR	0.001	0.023
FT3	PLR	0.002	0.002
FT4	RBC	<0.001	<0.001
FT4	Hemoglobin	<0.001	<0.001
FT4	Hematocrit	<0.001	<0.001
FT4	MCV	0.113	0.058
FT4	MCH	0.265	0.141
FT4	RDW	0.138	0.073
FT4	Platelets	0.006	0.004
FT4	MPV	0.032	0.013
FT4	WBC	0.042	0.802
FT4	NEU#	0.004	0.059
FT4	LYM#	0.073	0.037
FT4	MON#	0.146	0.410
FT4	NLR	0.002	0.002
FT4	PLR	0.507	0.507
lnTSH	RBC	0.135	0.123
lnTSH	Hemoglobin	0.010	0.005
lnTSH	Hematocrit	0.008	0.010
lnTSH	MCV	0.568	0.366
lnTSH	MCH	0.281	0.170
lnTSH	RDW	0.136	0.137
lnTSH	Platelets	0.244	0.276
lnTSH	MPV	0.054	0.022
lnTSH	WBC	0.003	0.038
lnTSH	NEU#	0.882	0.729
lnTSH	LYM#	<0.001	<0.001
lnTSH	MON#	0.003	0.381
lnTSH	NLR	0.157	0.386
lnTSH	PLR	0.001	0.134

Restricted cubic splines with four knots at the 5th, 35th, 65th, and 95th centiles of each exposure. Wald test on the non-linear spline terms, with HC3 robust standard errors, mutually adjusted for the other two thyroid analytes and adjusted for age and sex.

Table S3. Sensitivity analyses for the primary association

Sensitivity analysis	N	FT3 beta (95% CI)	P
Primary analysis	3673	+0.097 (+0.066 to +0.128)	<0.001
S1 Excluding thyroid-therapy biochemical proxies	3039	+0.074 (+0.041 to +0.107)	<0.001
S2 Strictly euthyroid only	2735	+0.085 (+0.050 to +0.120)	<0.001
S3 Outpatient encounters only	2717	+0.065 (+0.031 to +0.099)	<0.001
S4 Single-encounter records only	1593	+0.036 (-0.009 to +0.082)	0.113
S5a Women	2808	+0.108 (+0.071 to +0.145)	<0.001
S5b Men	865	+0.068 (-0.013 to +0.148)	0.098
S5c Age 18 to 50 years	2840	+0.072 (+0.038 to +0.107)	<0.001
S5d Age over 50 years	833	+0.137 (+0.070 to +0.205)	<0.001
S6a Additionally adjusted for MCV and RDW	3673	+0.086 (+0.059 to +0.113)	<0.001
S6b Additionally adjusted for WBC, NLR and platelets	3672	+0.090 (+0.059 to +0.122)	<0.001

S1 excluded records with a biochemical signature of thyroid hormone therapy. Medication records were unavailable. In S4 the smallest effect detectable at 80.0% power was 0.070, so that analysis is indeterminate rather than a failed replication.
Abbreviations: CI, confidence interval; FT3, free triiodothyronine.

Table S3 (addendum). Sensitivity analyses with additional adjustment for serum creatinine and vitamin B12, standardized β per 1 SD of FT3

Subset	n	Outcome	β before additional adjustment (95% CI)	p	β after additional adjustment (95% CI)	p
Records with a 2025 creatinine result	3201	Erythrocyte count	+0.090 (+0.056 to +0.123)	<0.001	+0.090 (+0.057 to +0.124)	<0.001
Records with a 2025 creatinine result	3201	Hemoglobin	+0.087 (+0.055 to +0.118)	<0.001	+0.089 (+0.058 to +0.120)	<0.001
Records with a 2025 vitamin B12 result	2363	Erythrocyte count	+0.087 (+0.049 to +0.125)	<0.001	+0.087 (+0.049 to +0.125)	<0.001
Records with a 2025 vitamin B12 result	2363	Hemoglobin	+0.085 (+0.050 to +0.120)	<0.001	+0.086 (+0.051 to +0.120)	<0.001
Records with both results	2099	Erythrocyte count	+0.081 (+0.040 to +0.122)	<0.001	+0.082 (+0.041 to +0.122)	<0.001
Records with both results	2099	Hemoglobin	+0.086 (+0.049 to +0.123)	<0.001	+0.089 (+0.052 to +0.126)	<0.001

Standardized β per 1 standard deviation (SD) of FT3 for the standardized outcome, mutually adjusted for FT4 and log TSH and adjusted for age and sex, with HC3 robust standard errors; restricted to FT3 within 3.5–6.5 pmol/L. "Before" models use the same records without the additional covariate(s); "after" models add log serum creatinine, log vitamin B12, or both. Creatinine and vitamin B12 results from 2025 were linked by medical record number from the same laboratory information system (median of the year's values per patient; results reported beyond an assay limit were taken at the reported limit; non-positive values were treated as missing). These analyses were added during peer review and were not pre-specified.
Abbreviations: CI, confidence interval; FT3, free triiodothyronine; FT4, free thyroxine; TSH, thyrotropin; SD, standard deviation.

Table S4. Iron indices and measured iron deficiency by red cell phenotype (primary cohort, linked subset)

Red cell phenotype	n (ferritin)	Ferritin, µg/L, median (IQR)	n (saturation)	Transferrin saturation, %, median (IQR)	Iron deficient, % (n/N)
Normocytic	852	25.9 (12.1–56.4)	316	20.5 (13.5–31.4)	33.3% (331/993)
Microcytic, acquired pattern	627	9.2 (4.5–21.6)	250	10.8 (6.6–19.0)	65.8% (482/733)
Microcytic, trait pattern	258	22.9 (8.4–57.9)	111	18.3 (8.3–28.9)	40.6% (129/318)

Iron indices linked by medical record number from the same laboratory information system for 2024–2025; median of available values per patient. Transferrin saturation = serum iron / total iron binding capacity × 100. Measured iron deficiency defined as transferrin saturation < 16% or ferritin < 15 µg/L. Kruskal–Wallis across the three strata: ferritin $p < 0.001$; transferrin saturation $p < 0.001$. Iron studies were requested selectively (anemia 51.3% in the linked subset versus 44.0% elsewhere), so this table validates the phenotype classification and is not part of the primary analysis. IQR, interquartile range.

Table S5. FT3 associations cross-classified by red cell phenotype and measured iron status (exploratory)

Group	n	Erythrocyte count, $\times 10^{12}$/L per pmol/L FT3	Hemoglobin, g/L per pmol/L FT3	MCH, pg per pmol/L FT3
Normocytic, iron replete	662	+0.111 (p = 0.001)	+3.56 (p < 0.001)	+0.065 (p = 0.596)
Normocytic, iron deficient	331	+0.031 (p = 0.429)	+0.87 (p = 0.395)	−0.008 (p = 0.962)
Acquired microcytic, iron deficient	482	+0.042 (p = 0.234)	+0.99 (p = 0.485)	0.000 (p = 1.000)
Trait pattern, iron replete	189	−0.051 (p = 0.424)	+1.19 (p = 0.468)	+0.418 (p = 0.116)

Unstandardized coefficients per 1 pmol/L FT3, mutually adjusted for FT4 and log TSH and adjusted for age and sex, HC3 robust standard errors. Iron deficiency = transferrin saturation < 16% or ferritin < 15 µg/L. The acquired-microcytic iron-replete and trait-pattern iron-deficient cells are omitted as too small or heterogeneous for interpretation. Iron studies were requested selectively; this table is exploratory and not part of the primary analysis.

Table S6. Association of free triiodothyronine with erythrocyte count and hemoglobin in the full ferritin-linked subset, the same-episode subset and the interval-bounded subset, with and without adjustment for log ferritin

Subset	n	Outcome and unit	Unadjusted for ferritin, β (95% CI)	p	Adjusted for log ferritin, β (95% CI)	p
Full ferritin-linked subset	1711	Erythrocyte count, $\times 10^{12}$/L per pmol/L FT3	+0.112 (+0.064 to +0.160)	<0.001	+0.111 (+0.063 to +0.159)	<0.001
Full ferritin-linked subset	1711	Hemoglobin, g/L per pmol/L FT3	+2.30 (+0.96 to +3.63)	0.001	+2.14 (+0.90 to +3.38)	0.001
Same-episode subset	894	Erythrocyte count, $\times 10^{12}$/L per pmol/L FT3	+0.094 (+0.033 to +0.155)	0.003	+0.093 (+0.032 to +0.154)	0.003
Same-episode subset	894	Hemoglobin, g/L per pmol/L FT3	+2.04 (+0.43 to +3.65)	0.013	+1.95 (+0.46 to +3.44)	0.010
Interval ≤30 days subset	992	Erythrocyte count, $\times 10^{12}$/L per pmol/L FT3	+0.102 (+0.042 to +0.162)	0.001	+0.100 (+0.040 to +0.159)	0.001
Interval ≤30 days subset	992	Hemoglobin, g/L per pmol/L FT3	+2.54 (+0.95 to +4.14)	0.002	+2.20 (+0.75 to +3.65)	0.003

Unstandardized coefficients per 1 pmol/L FT3, mutually adjusted for FT4 and log TSH and adjusted for age and sex, with HC3 robust standard errors; restricted to FT3 within 3.5–6.5 pmol/L. Full ferritin-linked subset: records with a numeric ferritin value (n = 1711). Same-episode subset: records with a single 2025 order date, in which ferritin and the index thyroid and blood count tests were drawn in the same laboratory episode (n = 894). Interval-bounded subset: the same-episode records plus records with several 2025 order dates whose span did not exceed 30 days (n = 992). The same-episode and interval-bounded analyses were added during peer review and were not pre-specified. Abbreviations: CI, confidence interval; FT3, free triiodothyronine; FT4, free thyroxine; TSH, thyrotropin.

Figure S1. Adjusted dose–response curves by red cell phenotype

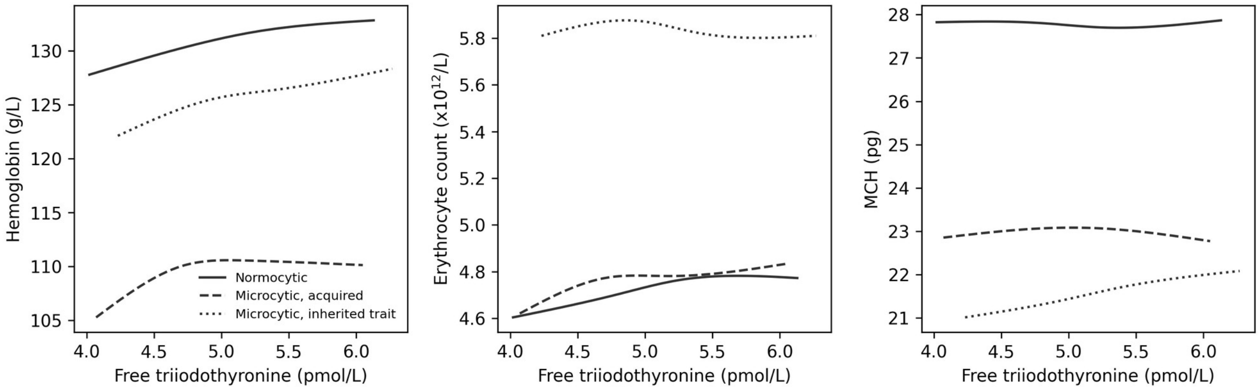

Figure S1. Adjusted dose–response curves for hemoglobin, erythrocyte count and mean corpuscular hemoglobin (MCH) across the free triiodothyronine (FT3) reference interval, by index-defined red cell phenotype (normocytic, solid line; microcytic acquired pattern, dashed line; microcytic presumed inherited-trait pattern, dotted line). Curves are fitted values from restricted cubic splines with four knots at the 5th, 35th, 65th and 95th centiles of FT3, mutually adjusted for free thyroxine and log thyrotropin and adjusted for age and sex (covariates held at their stratum means), with HC3 robust standard errors; each curve is drawn between the 5th and 95th centiles of FT3 in its stratum. The erythrocyte count curve rises and plateaus in the normocytic and acquired-pattern strata but is flat in the trait-pattern stratum, where the count is already high; the hemoglobin curves rise in all three strata.
